# Remediation of Toxic Heavy Metal Contaminated Soil by Combining a Washing Ejector Based on Hydrodynamic Cavitation and Soil Washing Process

**DOI:** 10.3390/ijerph19020786

**Published:** 2022-01-11

**Authors:** Hyunsoo Kim, Kanghee Cho, Oyunbileg Purev, Nagchoul Choi, Jaewon Lee

**Affiliations:** 1 Department of Energy and Resource Engineering, Chosun University, Gwangju 61452, Korea; star8538@naver.com (H.K.); oyunbileg@chosun.kr (O.P.); 2 Research Institute of Agriculture and Life Sciences, Seoul National University, Seoul 08826, Korea; nagchoul@snu.ac.kr; 3 JIU Corporation, Seoul 07528, Korea; jaewlee@jiuene.com

**Keywords:** washing ejector, cavitation, soil washing, phosphoric acid, heavy metals

## Abstract

Based on the features of hydrodynamic cavitation, in this study, we developed a washing ejector that utilizes a high-pressure water jet. The cavitating flow was utilized to remove fine particles from contaminated soil. The volume of the contaminants and total metal concentration could be correlated to the fine-particle distribution in the contaminated soil. These particles can combine with a variety of pollutants. In this study, physical separation and soil washing as a two-step soil remediation strategy were performed to remediate contaminated soils from the smelter. A washing ejector was employed for physical separation, whereas phosphoric acid was used as the washing agent. The particles containing toxic heavy metals were composed of metal phase encapsulated in phyllosilicates, and metal phase weakly bound to phyllosilicate surfaces. The washing ejector involves the removal of fine particles bound to coarse particles and the dispersion of soil aggregates. From these results we determined that physical separation using a washing ejector was effective for the treatment of contaminated soil. Phosphoric acid (H_3_PO_4_) was effective in extracting arsenic from contaminated soil in which arsenic was associated with amorphous iron oxides. Thus, the obtained results can provide useful information and technical support for field soil washing for the remediation of soil contaminated by toxic heavy metals through emissions from the mining and ore processing industries.

## 1. Introduction

Recently, contaminated soils in the vicinity of mines and smelters have become a matter of environmental concern in many countries. An increase in industrial activities, such as mining and smelting, has resulted in a broad range of environmental problems [1,2], in particular, the soil becoming contaminated by the accumulation of toxic heavy metals and metalloids [3]. These contaminants through emissions from the mining and ore processing industries are considered hazardous waste, causing local and diffuse pollution in the soil. In general, the soil at industrial sites is exposed to distinct groups of toxic heavy metal contaminants, which vary depending on the type of industry [4,5,6]. For example, soil at non-ferrous metal smelters are exposed to metal(loid)-rich, fine dust particles, which are primarily discharged with the smelting gases or ore dumps. Among the particulates, the metal-bearing particles were the most influential in the contaminated soil. These contaminants, containing a diverse form of toxic heavy metals, are associated with particles not heated up to melting temperatures, metal fragments and Fe oxide particles [7,8]. A number of previous studies have reported that contaminant emissions into the atmosphere on a regional scale could be related to wind direction and the size of particulates emitted by the smelter [9]. In particular, high levels of toxic heavy metals were found in topsoil. The fine metal particles in surface soil occurring in association with non-ferrous metal smelter activities include un-melted ore, unreacted flux and sulfide minerals (including As, Cu, Pb, Zn, etc.) [10].

Toxic heavy metals in contaminants can be slowly released by weathering and thereby co-precipitate metals as hydroxides and the occurrence of oxides of metals, such as Fe, Ti and Mn. Fe-bearing mineral phases are transformed into secondary metals species by the redox potential of the soil environment, Fe oxide is present in various forms such as hydrous oxides (e.g., ferrihydrite, hydrohematite, and maghemite), and oxyhydroxides (e.g., goethite, lepidocrocite, and feroxyhyte) [11,12,13]. In addition, the Fe oxide phase plays a key role in the amorphous toxic heavy metals sorption process because of their reactivity, surface area, and surface charge. Moreover, clay minerals have been correlated with adsorption and desorption of toxic heavy metals. Clay minerals belonging to the phyllosilicate group, are composed of tetrahedron and octahedron layered structures, which divide toxic heavy metals adsorption sites into surface, interlayer, and hydrate interlayer site [14]. Therefore, phyllosilicate-associated Fe oxide has been considered to be able to promote adsorption of amorphous toxic heavy metals. The soil properties, including organic and inorganic matter, are subject to sorption and precipitation, and toxic heavy metals can be retained in soil. Consequently, contaminated soil is related to the binding between the soil mineral particles and metal-bearing particles. Such binding in soil properties may have magnetic susceptibility. A soil’s magnetic properties, including organic, paramagnetic minerals, organometallics and ferro or ferric minerals, are divided into either positive or negative (diamagnetic) magnetic susceptibility [15].

Among the widely used physical, chemical, and biological soil remediation technologies, soil washing has been commonly used to remediate highly contaminated soil, owing to its speed and efficiency. Soil washing effectively reduces not only the concentration of the contaminants, but also the volume of the contaminated soil. Moreover, it involves a simple operation at a lower cost and, hence, can be applied to field remediation [16]. Typically, soil washing is based on the physicochemical reaction between the contaminants and the washing agents [17]. Therefore, selection of the washing agent is of critical importance for the soil washing process. Generally, inorganic acids, organic acids, and chelating agents are used as washing agents for the remediation of heavy metal contaminated soil. Particularly, organic and inorganic acids are known to achieve a much higher heavy metal extraction from contaminated soils [18,19]. However, these are ineffective in sites contaminated with both As and heavy metals, owing to their different characteristics. The remediation process is dependent on the chemical speciation with respect to the redox potential [20]. Particularly, the removal of As, most of which is strongly bound to Fe oxides, by soil washing has not been satisfactory. Most studies have focused on comparing the effects of experimental parameters (e.g., pH, soil/liquid (S/L) ratio, etc.) on heavy metal removal efficiency using various washing agents. Among these, while most investigations have focused on the efficiency of the heavy metal removal mechanisms, only a few have investigated the influence of soil characteristics on soil washing [21,22,23]. Since there is interest in minimizing negative effects of the washing treatment process on soil qualities [18], additional research is required for the development of an effective soil washing process. In particular, soil washing combined with physical separation can concentrate the metals into smaller soil volumes, resulting in effective heavy metal removal, thereby reducing the volume of the contaminated soil.

A washing ejector is a device that uses hydrodynamic cavitation. The cavitation flow of the hydrodynamic cavitation depends on the characteristics of the liquid pressure and kinetic energy [24,25]. We developed a washing ejector in this study that utilizes cavitation flow to remove fine particles in the contaminated soil. Through this research, we investigated the soil’s physicochemical properties, such as changes in contaminated soils between clay and Fe oxide. To accomplish this, the chemical and mineralogical characteristics of the contaminated soil were analyzed for their main properties and components. The main purpose of this study was to characterize the cavitation flow on the washing ejector and to separate fine particles of contaminated soil during the pretreatment using a washing ejector. Specifically, the objectives of this study are to find the optimal conditions of the pretreatment for the removal of As and heavy metals and to evaluate the characteristics of soil contaminants using the physicochemical and magnetic characterization. Furthermore, physical separation and soil washing as a two-step soil remediation strategy was performed to remediate contaminated soils.

## 2. Materials and Methods

### 2.1. Study Areas and Soil Characterization

Contaminated soil samples were collected from around an abandoned smelter location in Jang-hang, Korea. This site was widely contaminated with toxic heavy metals and was selected by the Korean government for reclamation activities as heavy metal contamination is a major environmental concern. This smelter was used to produce Cu and Pb products for approximately 60 years and was closed in 1989 [26]. Soil contamination occurred at this site due to scattering ores, refining residues, and chimney dust that contained heavy metals. Soil contamination revealed that the surrounding areas in a radius of up to approximately 4 km around the smelter were widely contaminated by heavy metals, including As, Cu, Pb, and Zn. The spatial distributions of pollutants in soils can be affected by prevailing wind directions, size of particulates, and distance from the source. Contaminants associated with particulates emitted from the smelting operations are concentrated in the ultrafine particle fraction [27]. The primary source of heavy metals at the smelter site was the deposition of smelter emission dust. The contaminated soil was collected approximately 2.5 km from the smelter site.

The soil samples used in this study were collected from a long-term contaminated site with a large area. We chose a surface layer soil sample (0–30 cm), which was then mixed, homogenized, air-dried, and ground before sieving to remove gravel with particle sizes > 2 mm. The results of the particle size analysis revealed the composition of sand (34.6%), silt (43.7%), and clay (21.7%), representing the textural classification of a loam soil. The observed primary physical and chemical properties of the soil were as follows: 6.40 pH, 2.74% organic matter, and 19.7 cmol/kg cation exchange capacity (CEC) (Table 1). The mineral composition analysis of the contaminated soil using XRD revealed that it consisted of sodium aluminum silicate, dickite, muscovite, and quartz.

### 2.2. A Washing Ejector and Experimental Procedure

**Figure 1:**
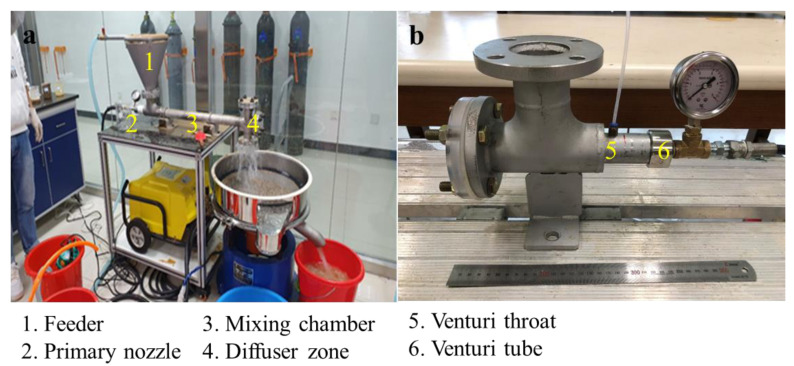
(**a**) The photo of the washing ejector and (**b**) the cavitating device.

A washing ejector is a pretreatment technology used to disperse soil aggregates by cavitation flow. In the washing ejector, the cavitation behavior is governed by the fluid velocity and pressure according to hydrodynamic cavitation. The washing ejector developed in this study is a device that utilizes fast liquid jets to remove pollutants. In these experiments, tap water was used as the fluid. Figure 1a shows a schematic diagram of the washing equipment on a small scale. The washing ejector comprises a feeder, a primary nozzle, a mixing chamber, and a diffuser zone. Figure 1b show the photographic details of the cavitating device and a nozzle. A nozzle was attached to a cavitating device connected to a washing ejector. The diameter of the Venturi tube (D) was 5 mm, and the throat diameter (d) was 3 mm, with a diameter ratio of 2.78. The dimensions of the Venturi tube chosen in this study were based on a numerical study on the optimization of geometrical parameters on the cavitation inception. The static pressure at the Venturi throat was measured using a digital pressure gauge (PX409-015GUSBH, Omega Engineering Inc., Norwalk, CT, USA). The outlet pressure remained constant and was equal to atmospheric pressure. The feeder was set at the top of the chamber, and the nozzle was parallel to the axis of the washing ejector. The fluid was sprayed from the cavitating device into the mixing chamber zone, where soil was completely mixed via the feeder, and then sieved by vibrating a 0.053 mm screen instrument. The study soil is made up of a mixture of natural aggregates. In particular, the fine particles containing toxic heavy metals were composed of metal phase included in phyllosilicates and metal phase weakly bound on surface phyllosilicates. The fine clay minerals were removed by a washing ejector. The cut off size of the treated soil was set as size 0.053 mm considering the loss of fine particles.

In general, As is often absorbed by Fe oxides, which form the most important and widely distributed magnetic minerals within soils. These particulates can act as carriers of metals through adsorption and incorporation into their crystalline structures. Thus, fine particles are responsible for toxic heavy metal accumulation in soil. After the treatment with the washing ejector, the magnetic properties of the washed soil were observed using a vibrating sample magnetometer (VSM, LakeShore 7407-S, Lake Shore Cryotronics, Inc., Westerville, OH, USA).

### 2.3. Removal of Toxic Heavy Metals with Phosphoric Acid

Toxic heavy metal removal in the contaminated soil was related to the washing conditions, metal fraction distribution, and soil properties. The effectiveness of phosphoric acid in improving the efficiency of toxic heavy metal extraction has been the subject of many previous studies [19]. The experiments were conducted with various phosphoric acid concentrations to extract toxic heavy metals from treated soil by using a washing ejector. The batch experimental conditions were determined based on a previous study, wherein they were successfully applied to extract toxic heavy metals from the contaminated soil [28]. Soil washing experiments were performed with 5 g of soil with 35 mL of washing solution in a 50 mL conical tube. The suspensions were then shaken at 250 rpm for 2 h at 20 °C in a shaking incubator. Each experiment was performed in duplicate and repeated at least twice. The relative standard deviations (*n* = 2) of the duplicates were less than 10%.

### 2.4. Analysis Method

The pH of the soil sample was analyzed by mixing with deionized water at a ratio of 1:5 (soil/deionized water). EPA method 9081 was used to analyze the CEC of the soil, and the organic matter content was determined by the ignition method (weight loss at 450 °C). The oversize materials (stones) were separated from the soil by sieving through a 4 mm screen. For the separation procedure, the air-dried sample (300 g) was weighed and dry-sieved. Then, 300 g of soil was placed on top of a nest of sieves and fractionated into seven aggregate sizes using a vibrating screen instrument. The distribution of the soil was determined by the weight of each size after sieving.

A polished section of the soil was examined using a microscope (ECLIPSE LV100DOL, Nikon, Tokyo, Japan). Polished sections were prepared by placing soil in an epoxy resin, which, after curing, was polished to ensure flatness. The morphology and surface structure of the soil samples were analyzed by field emission SEM (FE-SEM, S4800, Hitachi, Tokyo, Japan) with an EDS (ISIS310, Jeol, Tokyo, Japan). The samples were subjected to XRD (X’Pert Pro MRD, Panalytical, Amsterdam, The Netherlands). Cu Kα radiation was used at an acceleration voltage of 40 kV and a current of 30 mA. The 2θ section from 10° to 70° was analyzed for the soil. Fourier-transform infrared (FTIR) spectroscopy (Nicolet 6700, Thermo Fisher Scientific, Waltham, MA, USA) was used to analyze the soil.

The total heavy metal concentrations were determined in both bulk and washed samples by atomic-absorption spectrophotometry (AAS, AA-7000, Shimadzu, Kyoto, Japan). The heavy metals in the soil were measured based on the Korean Standard Test methods (aqua regia) and compared to the Korean warning standards for forest land and residential areas. The total concentrations of heavy metals in 1 g soil samples were extracted using HCl and HNO_3_ at a 3:1 ratio (i.e., aqua regia) and the extracts were filtered for analysis. The chemical forms of As in the samples were analyzed using the sequential extraction procedure described by Wenzel et al. (2001), which divides As-bound soil fractions into five fractions. The fractionations of Cu and Pb in the soil were also determined using a traditional sequential extraction method (i.e., Tessier’s method) for the standards and measurements. The detailed conditions of the sequential extraction procedures are summarized in Table A1. After each extraction step, the samples were centrifuged at 3000× *g* rpm for 10 min, filtered, and analyzed via AAS.

## 3. Results and Discussion

### 3.1. A Washing Ejector Based on Hydrodynamic Cavitation

**Figure 2:**
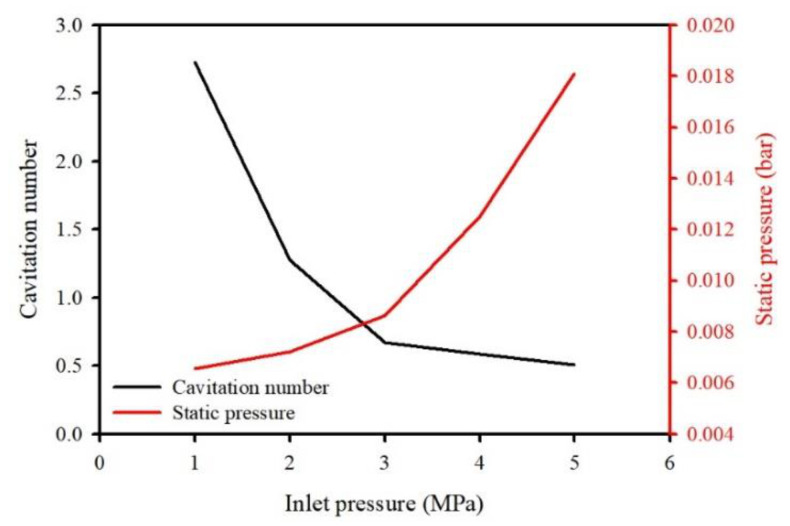
The hydrodynamic condition presented as the effect of inlet pressure on cavitation number and static pressure.

To evaluate the characteristics of the cavitating flow using the washing ejector, the inlet pressure was controlled using the flow control valves, ranging from 1 to 5 MPa gauge pressure. The cavitation phenomenon is related to the pressure drop in a flowing liquid through the Venturi tube. During this process, air was sucked into the cavitating device (i.e., the Venturi throat) in the washing ejector, indicating that the intensity of cavitation flow increases with an ascending pressure drop in the Venturi throat. The flow rate was 1.3 L/min for inlet pressure of 1 MPa, 2.6 L/min for 3 MPa and 3.0 L/min for 5 MPa. With the increase in the inlet pressure, the static pressure difference increased from 0.007 bar to 0.018 bar (Figure 2). The cavitating flow can be generated by alterations in pressure, which are caused by specific constructions such as Venturi tubes. In this respect, the cavitation number is derived from Bernoulli’s theorem and plays a crucial role in the inception of cavitation [26]. The cavitation number (C_v_) is a dimensionless parameter that represents the cavitation intensity. The formula for the cavitation number is expressed as (1):

(1) $$C_{v}=\frac{P_{2}-P_{v}}{\frac{1}{2}\rho V_{0}^{2}}$$

where P_2_ is the fully recovered downstream pressure, P_v_ is the vapor pressure of the liquid at the reference temperature, ρ is the density of the liquid, and V_0_ is the velocity of the liquid at the Venturi throat.

**Figure 3:**
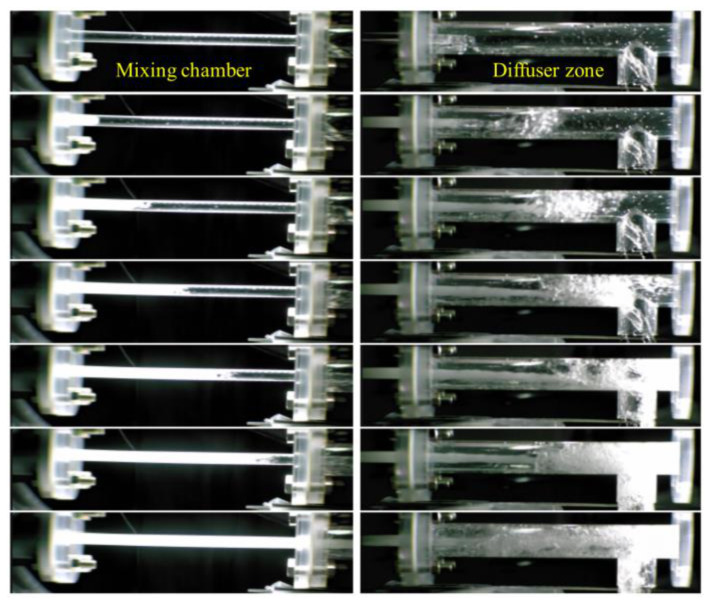
Flow visualization of cavitation flow generation resulting from the washing ejector at 5 MPa.

Eventually, the static pressure affected the cavitating flow in the washing ejector. As can be seen, the gauge pressure ascends as the static pressure difference increases and the cavitation number was gradually decreased. These results demonstrated that the static pressure ascending could promote the inception of cavitation and the cavitation number is a suitable estimate of cavitating flow. The cavitation generation point corresponds to C_v_ = 1, and cavitation occurs when C_v_ < 1. The experimental results showed reasonable agreement with the CFD results for cavitation behaviors published in the literature [29,30,31]. Therefore, in this study, the fluid pressure was 5 MPa using the characteristics of the cavitating flow. Figure 3 presents the visualization of cavitating flow generation in the washing ejector.

### 3.2. Characterization of the Contaminated Soil

**Figure 4:**
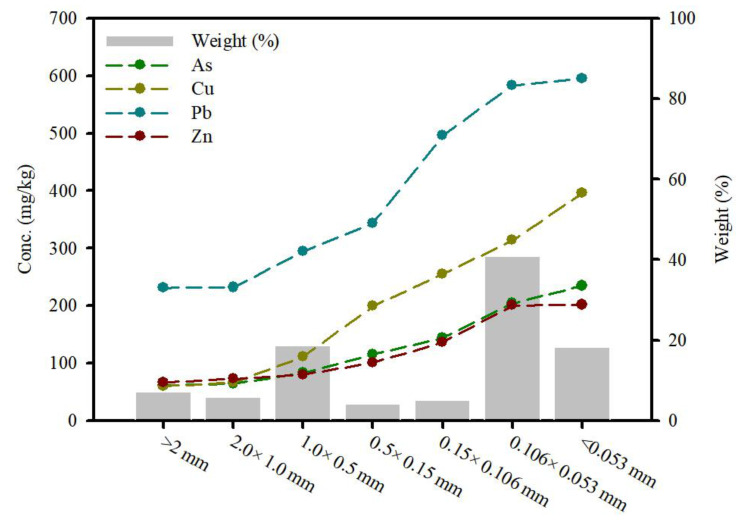
Concentration distribution of toxic heavy metals (mg/kg) and weight (%) according to particle size.

The concentrations of toxic heavy metals in the contaminated soil are summarized in Table 1. The samples were contaminated with As, Cu, and Pb and their concentrations were 25 mg/kg, 150 mg/kg, and 200 mg/kg, respectively, which exceeded the cleanup level of the Korean Soil Environment Conservation Act (KSECA). The Zn concentrations of the soils were below the levels of concern of KSECA (300 mg/kg). A size separation study was conducted in order to measure the contaminant concentration in the soils with different particle sizes (Figure 4). The results demonstrate that the finer soil particles had higher toxic heavy metal concentrations compared to the coarser soil particles. This is because fine soil particles have greater sorption capacities due to their larger specific surface area. Therefore, the particle size analysis demonstrates that contaminants adsorb on clay and silt to a greater degree compared to sand. The soil particles were divided into seven fraction sizes, >2.0 mm, 2–1 mm, 1–0.5 mm, 0.5–0.15 mm, 0.15–0.106 mm, 0.106–0.053 mm, and <0.053 mm. The distribution of the soil was determined by the weight of each sieve after sieving, which corresponded to 7.17%, 5.87%, 18.6%, 4.16%, 5.00%, 40.9%, and 18.3%, respectively, of the bulk soil sample. Overall, the distribution characteristics were significantly related to the particle size and the contaminated soil concentration.

**Figure 5:**
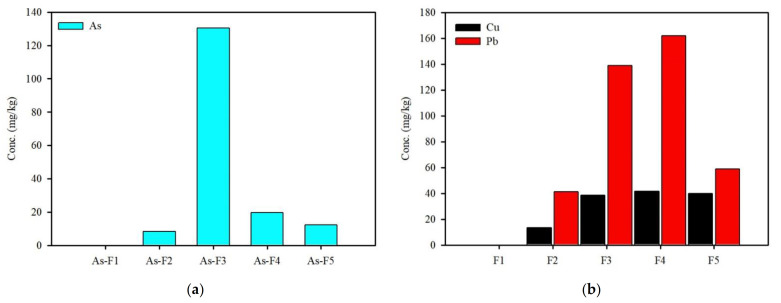
Sequentially extracted metal fractions from the contaminated soil. For As, each fraction represents: (**a**) (As-F1) non-specifically sorbed; (As-F2) specifically sorbed; (As-F3) amorphous oxide-associated; (As-F4) crystalline oxide-associated; (As-F5) residual. For Cu and Pb, each fraction represents (**b**) (F1) exchangeable; (F2) carbonate; (F3) amorphous Fe and Mn hydroxide; (F4) organic matter bound and sulfide; (F5) residual.

A five-step sequential extraction of As in the soil samples was conducted as described by Wenzel et al. (2001) (Figure 5a). Sequential extraction showed that the quantities of the five fractions of soils were in the order of F3 >> F4 > F5 > F2 > F1. The As in the sample was mostly bound to amorphous oxides (76.4%, F3), of which a relatively lower proportion (<5.0%) was non-specifically/specifically adsorbed onto the mineral surface. The As extracted with crystalline oxides (F4) was the second most dominant phase, comprising 11.5% of the total As. The sequential extraction analysis implied that the contaminant contained As in various forms, not limited to the discrete mineral phase but including As incorporation into the structure of phyllosilicates or Fe-Mn (oxy) hydroxides. Adsorption of As in soil occurs mainly on Fe-oxides rather than organic matter. Sequential extractions (Tessier’s method) were also conducted to evaluate the Cu and Pb speciation in the soil (Figure 5b). The Cu and Pb in the soil were associated with Fe-Mn oxides (Cu, 39.8% and Pb, 30.4%, Step 3), organic matter (Cu, 34.9% and Pb, 51.2%, Step 4), and residual (Cu, 15.7% and Pb, 12.5%, Step 5) fractions. The high fractions of heavy metals extracted in Steps 3 and 4 from the contaminated soil indicate that the metals were strongly associated with the minerals in the soil.

**Figure 6:**
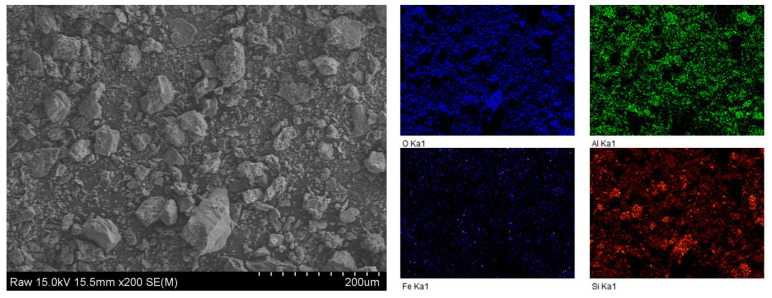
FE-SEM micrograph and corresponding EDS elemental mapping of the contaminated soil.

The SEM-EDS analysis of the metal(loid)-bearing particles from the bulk samples showed that the soil particles were composed of silicate grains surrounded by an amorphous oxide (Figure 6). EDS mapping showed that the soil contained high portions of Al, Si, and O, as phyllosilicates are a basic component of soil. High levels of Fe were observed. However, the low detection levels of As, Cu, and Pb-containing particles, despite the high concentration of As, Cu, and Pb in the soil, implied the presence of As, Cu, and Pb in the soil in various forms, which could not be observed by SEM-EDS because they were not present in mineral phases.

**Figure 7:**
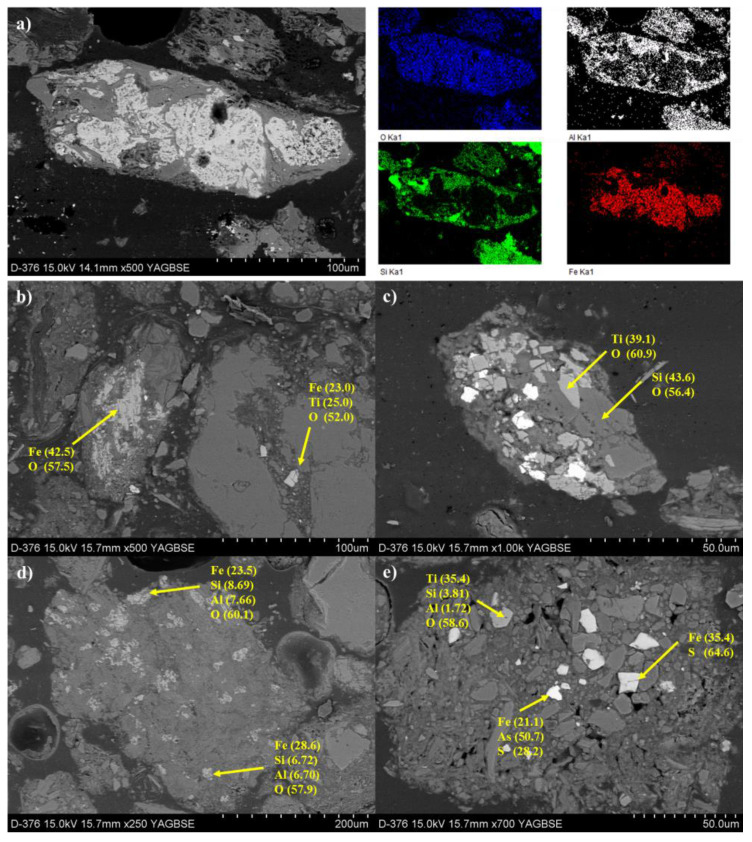
Scanning electron micrographs in backscattered electrons (BSE) of polished sample. (**a**) Scanning electron micrographs in backscattered electrons (BSE) and EDS mapping. (**b**–**e**) Back-scattered electron (BSE) image and EDS spot analysis. Element concentrations are expressed in wt%.

In the microscopic image of the polished section, the mineral phases were observed in the form of quartz, Ti oxide, and Fe oxides, such as hematite and magnetite. Soil mineralogy is a combination of primary components (i.e., phyllosilicate) and secondary phases produced by industrial activities and weathering processes. Secondary phases sizes range from <1 μm to about 30 μm. The microstructure of the polished section, investigated through SEM-EDS analysis, was composed of Fe oxides embedded within the silicate matrix. Fe oxide was visible in back-scattered electron (BSE) images as a white halo on light gray particles. The elemental mapping by EDS in Figure 7a shows four different compositional regions of Al, Si, Fe, and O. The focus on the zones with light gray particles indicate that Fe- and Ti-oxides impregnated the space of the silicate grains (Figure 7b–e). These secondary phases were present as discrete euhedral to subhedral grains with irregularly shaped morphologies. According to the elemental composition analysis of the individual light gray particles, the minerals within the silicate matrix can be classified into the following groups: Fe-O, Fe-Ti-O, Ti-O, Al-Si-O, and Fe-As-S. Minerals associated with Al, Si, Ti, and Fe were characterized as silicate minerals and Fe and Ti-oxides. The SEM images reveal the variety of metallic oxide phases identified in the soil. These phases might release metals when subjected to weathering processes or during water–soil interaction.

### 3.3. Effect of Soil Separation Using a Washing Ejector

**Figure 8:**
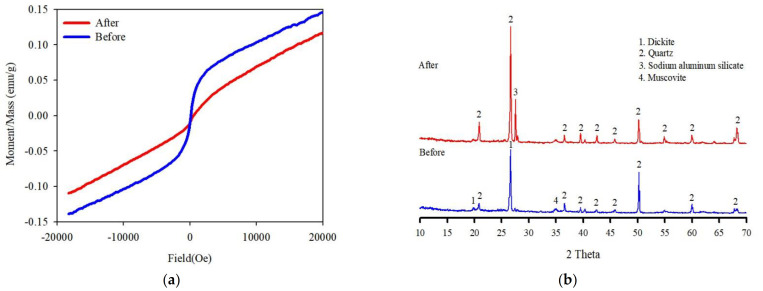
(**a**) Hysteresis loop and (**b**) XRD patterns of the original soil and treated soil.

All experiments were preceded by a washing ejector using the characteristics of the cavitating flow. Soil and water were mixed in a washing ejector with a mass ratio of 1:2. After the pretreatment, the mixture was separated by a vibrating screen (0.053 mm) to obtain treated soil. The results of the magnetic susceptibility measurements at room temperature displayed differences depending on both before and after the treatment with the washing ejector. The saturation magnetizations of before and after treatment were found to be approximately 0.15 and 0.12 emug^−1^, respectively (Figure 8a). The magnetic properties of the soil before processing may be due to the presence of a relatively large amount of clay and fine soil particles. Figure 8b presents XRD patterns that show variation in the structure of the phyllosilicate in the treated soil with the washing ejector. These results clearly show that the cavitating flow effectively removed the fine particle fractions of the soil. Therefore, the variations in quartz peak intensities indicate that the grains surrounded by clay minerals containing Fe oxide were liberated. Moreover, this is due to the existence of a large amount of diamagnetic materials, such as quartz.

**Figure 9:**
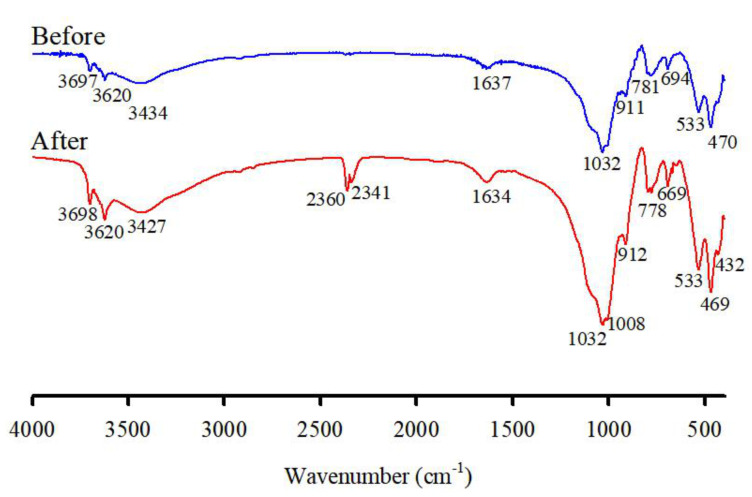
FT-IR spectra of the original soil (before) and treated soil (after) by a washing ejector.

The obtained infrared spectroscopy spectra of the soil showed the characteristic bands of its main functional groups (Figure 9). The main groups are the OH groups, which are mainly structural groups in the soil, adsorbed water molecules, stretched carboxyl group groups, Si-O stretching, Si-O-Si stretching, and Si-O-Al stretching [32,33]. The characteristic bands of 3697, and 3620 cm^−1^ correspond to the stretching vibration of the hydroxyl groups (OH) of Si and Al, respectively, in the clay minerals. Meanwhile, the absorption peaks at 1637 cm^−1^ can be attributed to the C=O stretching vibration of the carboxyl group (-COO) [7,8]. The Si-O-Si band is observed at 1032 cm^−1^ as a result of the Si-O vibration. The band at 911 cm^−1^ corresponds to the OH deformation of hydroxyl groups. The Si-O bending vibration modes at 694 and 781 cm^−1^ are characteristic frequencies of quartz.

After treatment with the washing ejector, the representative bands of treated soil functional groups appeared in deviated positions, confirming changes in their structural properties. The FTIR spectra revealed an increase in the intensity of the main bands, and a new band of hydroxyl groups, Si-O-Al and Si-O-Si, appeared at 1008 cm^−1^, 912 cm^−1^, 796 cm^−1^, 778 cm^−1^, 693 cm^−1^, 669 cm^−1^, 649 cm^−1^, 533 cm^−1^, and 469 cm^−1^ as a result of physical separation. In particular, the main alterations in the structural properties were represented by an intense band at 2360 cm^−1^ that was attributed to the C=O asymmetric stretching vibration, due to atmospheric CO_2_.

### 3.4. Removal of Toxic Heavy Metals with Phosphoric Acid

**Figure 10:**
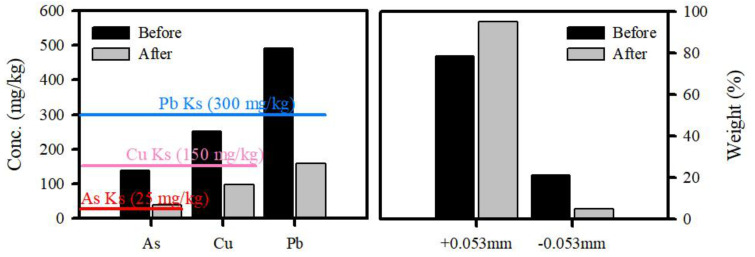
The toxic heavy metal concentrations (mg/kg) in the original soil (before) and treated soil (after) and recovery weight (%) of soil by a washing ejector.

A washing ejector can be effective in reducing the removal of fine particles. The results show that the efficiency of toxic heavy metals removal greatly improved (Figure 10). The treated soil concentration decreased from 139.5 to 39.3 mg/kg (As), 252.4 to 98.5 mg/kg (Cu), and 490.7 to 158.6 mg/kg (Pb). The residual concentrations of toxic heavy metals, including Cu and Pb were below the cleanup level of KSECA. Notably, a greater amount of fine fractions (−0.053 mm) was observed in the original soil sample (i.e., untreated soil) than in the treated soil, implying that more fine particles were bound to particles in the original soil.

**Figure 11:**
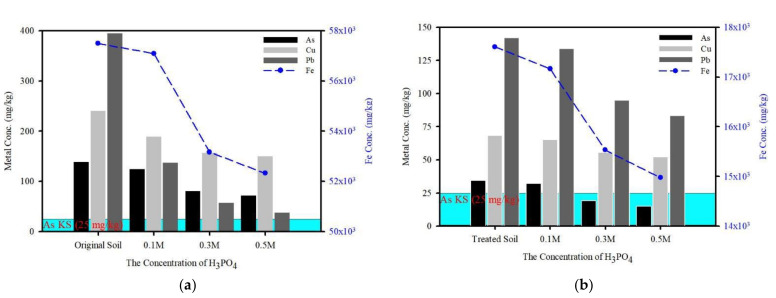
Effect of phosphoric acid concentrations on enhanced toxic heavy metals extractions from the original soil (**a**) and treated soil (**b**).

In order to study the effect of phosphoric acid on the amount of fine particles using a washing ejector, different concentrations were applied to the original soil and treated soil, and the extracted As, Cu, Fe, and Pb increased with increasing phosphoric acid concentration (Figure 11). The As in the original soil was largely associated with amorphous (Fraction 3) and crystalline oxides (Fraction 4) by sequential extraction. Total Fe concentrations in the original soil and treated soil were 57,490 and 17,605 mg/kg, respectively. The original soil had a higher proportion of Fe oxides compared to treated soil, whilst similar extraction trends of As and Fe were observed. Therefore, this indicates that adsorption of As in soil is correlated with Fe oxides. Specifically, the release of As was likely due to the extraction of Fe oxides. As a result, As remained present in the original soil despite the increasing phosphoric acid concentration. Therefore, it is likely that Fe oxide was predominantly encapsulated in soil components between the phyllosilicate’s surface and layer.

**Figure 12:**
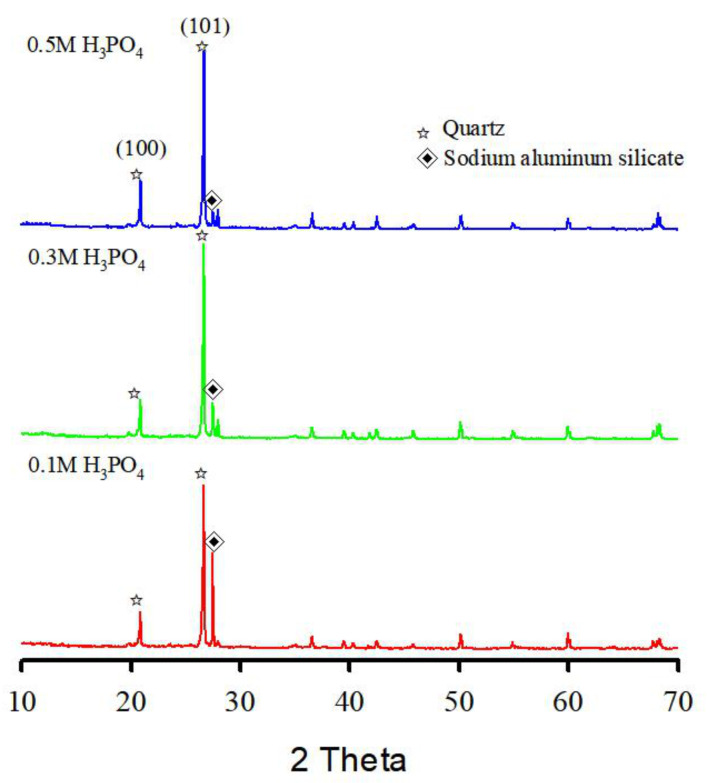
X-ray diffraction patterns for residual solid after soil washing by phosphoric acid (H_3_PO_4_).

Phosphoric acid is polyprotic and can release more H^+^ (Equations (2)–(4)), which is conducive to the removal of cationic metals under acidic conditions. The soil samples washed with phosphoric acid were characterized by XRD (Figure 12). After soil washing, the XRD patterns of the washed soil showed that sodium aluminum silicate and quartz peaks were present as the (100) and (101) plane intensity increased compared with before the reaction of the treated soil. However, when the washed soil reacted with phosphoric acid, the sodium aluminum silicate peak intensity decreased with increasing phosphoric acid concentration. As the consumption of phosphoric acid continued, the amorphous toxic heavy metal bound to the phyllosilicate surfaces was extracted, which led to the dissolution of sodium aluminum silicate (process described in Equation (5)). Consequently, the washing ejector demonstrates that the soil particles containing toxic heavy metals are predominantly composed of amorphous toxic heavy metal bound to the phyllosilicate surfaces rather than encapsulated in the phyllosilicates.

(2) $$H_{3}PO_{4}\;⇌\;H^{+}+H_{2}PO_{4}^{-}$$

(3) $$H_{2}PO_{4}^{-}\;⇌\;H^{+}+HPO_{4}^{2-}$$

(4) $$HPO_{4}^{-}\;⇌\;H^{+}+PO_{4}^{-}$$

(5) $$NaAlSiO_{4}+4H^{+}+PO_{4}^{-}⇌\;Na^{+}+AlPO_{4}+2H_{4}SiO_{4\left(aq\right)}$$

## 4. Conclusions

Soils are contaminated by the accumulation of toxic heavy metals emitted to the atmosphere by smelters. The contaminated surface soil sample used in this study showed that the concentrations of toxic heavy metals such as As, Cu, and Pb exceeded the cleanup level of KSECA. The mineralogical constituents of soil are predominantly silicate minerals. The particles containing toxic heavy metals were composed of metal phase encapsulated in phyllosilicates, and metal phase weakly bound on surface phyllosilicates. Based on the features of hydrodynamic cavitation, the device developed in this study utilizes cavitating flow. 

In the present study, we used a dimensionless parameter to characterize the working conditions of a washing ejector for the removal of fine particles. The liberation of the discrete minerals was increased due to the washing ejector. Therefore, a greater amount of fine fractions was observed in the original soil sample than in the treated soil, implying that more fine particles were bound to particles in the original soil. Overall, the soil organic matter of several functional groups (carboxyl and hydroxyl) could be liberated, resulting in the detachment of clay minerals or amorphous oxides, increasing the quartz peaks. Phosphoric acid could enhance the release of toxic heavy metals in soil after the physical separation. The results of this study can be of use in the remediation of soils contaminated with toxic heavy metals.

## Figures and Tables

**Table 1:** Physicochemical characteristics of the contaminated soil.

Soil Characteristic	Values	Contaminants	Values (mg/kg)	Regulation Level in Korea ^1^ (mg/kg)
pH	8.85	As	139.53 ± 2.65	25
CEC (cmol/kg)	4.25	Cu	252.40 ± 2.55	150
Organic matter (%)	27.6	Pb	490.74 ± 3.55	200
Texture	Loam	Zn	135.37 ± 3.58	300

^1^ Concerning level of the Soil Environment Conservation Act of Korea (KSECA) legislated by Korean Ministry of Environment (K-MOE).

## Data Availability

All data generated or analyzed during this study are included in this published article.

## Appendix A

**Table A1:** Extraction procedures of two sequential extraction procedures.

**Step (Wenzel et al., 2001)**	**Extractable Phase**	**Extraction Conditions**
F1	Non-specially bound	0.05 M (NH_4_)_2_SO_4_
F2	Specially bound	0.05 M (NH_4_)H_2_PO_4_
F3	Amorphous hydrous oxides of Fe and Al bound	0.2 M NH_4_-oxalate buffer; pH 3.25
F4	Crystalline hydrous oxides of Fe and Al bound	0.2 M NH_4_-oxalate buffer + 0.1 M ascorbic acid; pH 3.25
F5	Residual	18 mL HNO_3_+ 8 mL HF + 2mL H_2_O_2_+ 2mL H_2_O
**Step (Tessier et al., 1979)**	**Extractable Phase**	**Extraction Conditions**
F1	Exchangeable	1 M MgCl_2_; pH 7.0
F2	Bound to carbonate	1 M NaOAc; pH 5.0
F3	Bound to amorphous Fe and Mn hydroxides	0.04 M NH_2_OH-HCl in 25% (*v*/*v*) HOAc
F4	Bound to organic matters and sulfides	0.02 M HNO_3_ + 5 mL of 30% H_2_O_2_; pH 2.0, 30% H_2_O_2_; pH 2, 3.2 M NH_4_OAc in 20% (*v*/*v*) HN03
F5	Residual	HClO_4_ (2 mL) + HF (10 mL), HClO_4_ (1 mL) + HF (10 mL), HClO_4_ (1 mL), 12 N HCl

## References

[1] Lee P.-K., Yu S., Jeong Y.-J., Seo J., Choi S.G., Yoon B.-Y. (2019). Source identification of arsenic contamination in agricultural soils surrounding a closed Cu smelter, South Korea. Chemosphere.

[2] Kang M.-J., Kwon Y.K., Yu S., Lee P.-K., Park H.-S., Song N. (2019). Assessment of Zn pollution sources and apportionment in agricultural soils impacted by a Zn smelter in South Korea. J. Hazard. Mater..

[3] Csavina J., Field J., Taylor M.P., Gao S., Landázuri A., Betterton E.A., Sáez A.E. (2012). A review on the importance of metals and metalloids in atmospheric dust and aerosol from mining operations. Sci. Total Environ..

[4] Shin W., Choung S., Han W.S., Hwang J., Kang G. (2018). Evaluation of multiple PRPs’ contributions to soil contamination in reclaimed sites around an abandoned smelter. Sci. Total Environ..

[5] Lee P.-K., Yu S., Chang H.J., Cho H.Y., Kang M.-J., Chae B.-G. (2016). Lead chromate detected as a source of atmospheric Pb and Cr (VI) pollution. Sci. Rep..

[6] Ettler V. (2016). Soil contamination near non-ferrous metal smelters: A review. Appl. Geochem..

[7] Sierra C., Martínez J., Menéndez-Aguado J.M., Afif E., Gallego J. (2013). High intensity magnetic separation for the clean-up of a site polluted by lead metallurgy. J. Hazard. Mater..

[8] Van Groeningen N., ThomasArrigo L.K., Byrne J.M., Kappler A., Christl I., Kretzschmar R. (2020). Interactions of ferrous iron with clay mineral surfaces during sorption and subsequent oxidation. Environ. Sci. Process. Impacts.

[9] Lee P.-K., Kang M.-J., Yu S., Kwon Y.K. (2020). Assessment of trace metal pollution in roof dusts and soils near a large Zn smelter. Sci. Total Environ..

[10] Lee P.-K., Kang M.-J., Jeong Y.-J., Kwon Y.K., Yu S. (2020). Lead isotopes combined with geochemical and mineralogical analyses for source identification of arsenic in agricultural soils surrounding a zinc smelter. J. Hazard. Mater..

[11] Ettler V., Cihlová M., Jarošíková A., Mihaljevič M., Drahota P., Kříbek B., Vaněk A., Penížek V., Sracek O., Klementová M. (2019). Oral bioaccessibility of metal (loid) s in dust materials from mining areas of northern Namibia. Environ. Int..

[12] Contessi S., Calgaro L., Dalconi M.C., Bonetto A., Bellotto M.P., Ferrari G., Marcomini A., Artioli G. (2020). Stabilization of lead contaminated soil with traditional and alternative binders. J. Hazard. Mater..

[13] Portillo H., Zuluaga M.C., Ortega L.A., Alonso-Olazabal A., Murelaga X., Martinez-Salcedo A. (2018). XRD, SEM/EDX and micro-Raman spectroscopy for mineralogical and chemical characterization of iron slags from the Roman archaeological site of Forua (Biscay, North Spain). Microchem. J..

[14] Abukhadra M.R., Mostafa M. (2019). Effective decontamination of phosphate and ammonium utilizing novel muscovite/phillipsite composite; equilibrium investigation and realistic application. Sci. Total Environ..

[15] Uddin M.K. (2017). A review on the adsorption of heavy metals by clay minerals, with special focus on the past decade. Chem. Eng. J..

[16] Li Y., Liao X., Li W. (2019). Combined sieving and washing of multi-metal-contaminated soils using remediation equipment: A pilot-scale demonstration. J. Clean. Prod..

[17] Jho E.H., Im J., Yang K., Kim Y.-J., Nam K. (2015). Changes in soil toxicity by phosphate-aided soil washing: Effect of soil characteristics, chemical forms of arsenic, and cations in washing solutions. Chemosphere.

[18] Im J., Yang K., Jho E.H., Nam K. (2015). Effect of different soil washing solutions on bioavailability of residual arsenic in soils and soil properties. Chemosphere.

[19] Deng B., Li G., Luo J., Ye Q., Liu M., Rao M., Jiang T., Bauman L., Zhao B. (2019). Selectively leaching the iron-removed bauxite residues with phosphoric acid for enrichment of rare earth elements. Sep. Purif. Technol..

[20] Kim E.J., Jeon E.-K., Baek K. (2016). Role of reducing agent in extraction of arsenic and heavy metals from soils by use of EDTA. Chemosphere.

[21] Andreozzi R., Fabbricino M., Ferraro A., Lerza S., Marotta R., Pirozzi F., Race M. (2020). Simultaneous removal of Cr(III) from high contaminated soil and recovery of lactic acid from the spent solution. J. Environ. Manag..

[22] Bianco F., Race M., Papirio S., Oleszczuk P., Esposito G. (2021). The addition of biochar as a sustainable strategy for the remediation of PAH-contaminated sediments. Chemosphere.

[23] Kominkova D., Fabbricino M., Gurung B., Race M., Tritto C., Ponzo A. (2018). Sequential application of soil washing and phytoremediation in the land of fires. J. Environ. Manag..

[24] Shi H., Li M., Nikrityuk P., Liu Q. (2019). Experimental and numerical study of cavitation flows in venturi tubes: From CFD to an empirical model. Chem. Eng. Sci..

[25] Sarvothaman V.P., Simpson A.T., Ranade V.V. (2019). Modelling of vortex based hydrodynamic cavitation reactors. Chem. Eng. J..

[26] Lim S.-Y., Min S.-H., Yoo S.-H. (2016). The public value of contaminated soil remediation in Janghang copper smelter of Korea. Res. Policy.

[27] Jeong S., Hong J.K., Jho E.H., Nam K. (2019). Interaction among soil physicochemical properties, bacterial community structure, and arsenic contamination: Clay-induced change in long-term arsenic contaminated soils. J. Hazard. Mater..

[28] Cho K., Myung E., Kim H., Park C., Choi N., Park C. (2020). Effect of Soil Washing Solutions on Simultaneous Removal of Heavy Metals and Arsenic from Contaminated Soil. Int. J. Environ. Res. Public Health.

[29] He D., Bai B. (2012). Numerical investigation of wet gas flow in Venturi meter. Flow Meas. Instrum..

[30] Badve M., Gogate P., Pandit A., Csoka L. (2013). Hydrodynamic cavitation as a novel approach for wastewater treatment in wood finishing industry. Sep. Purif. Technol..

[31] Petkovšek M., Mlakar M., Levstek M., Stražar M., Širok B., Dular M. (2015). A novel rotation generator of hydrodynamic cavitation for waste-activated sludge disintegration. Ultrason. Sonochem..

[32] Kim E.J., Baek K. (2015). Enhanced reductive extraction of arsenic from contaminated soils by a combination of dithionite and oxalate. J. Hazard. Mater..

[33] Nejad Z.D., Jung M.C., Kim K.-H. (2018). Remediation of soils contaminated with heavy metals with an emphasis on immobilization technology. Environ. Geochem. Health.

